# Transgenerational inheritance of enhanced susceptibility to radiation-induced medulloblastoma in newborn *Ptch1^+/−^* mice after paternal irradiation

**DOI:** 10.18632/oncotarget.5553

**Published:** 2015-10-03

**Authors:** Lorena Paris, Paola Giardullo, Simona Leonardi, Barbara Tanno, Roberta Meschini, Eugenia Cordelli, Barbara Benassi, Maria Grazia Longobardi, Alberto Izzotti, Alessandra Pulliero, Mariateresa Mancuso, Francesca Pacchierotti

**Affiliations:** ^1^ Division of Health Protection Technologies, Agenzia Nazionale per le Nuove Tecnologie, l'Energia e lo Sviluppo Economico Sostenibile (ENEA), Rome, Italy; ^2^ Department of Ecological and Biological Sciences, Tuscia University, Viterbo, Italy; ^3^ Department of Radiation Physics, Guglielmo Marconi University, Rome, Italy; ^4^ Department of Sciences, University of Roma Tre, Rome, Italy; ^5^ Department of Health Sciences, University of Genoa, Genoa, Italy; ^6^ IRCCS AOU San Martino IST Genoa, Italy

**Keywords:** transgenerational carcinogenesis, epigenetic inheritance, medulloblastoma, patched1 knockout mice, microRNA

## Abstract

The hypothesis of transgenerational induction of increased cancer susceptibility after paternal radiation exposure has long been controversial because of inconsistent results and the lack of a mechanistic interpretation. Here, exploiting *Ptch1* heterozygous knockout mice, susceptible to spontaneous and radiation-induced medulloblastoma, we show that exposure of paternal germ cells to 1 Gy X-rays, at the spermatogonial stage, increased by a considerable 1.4-fold the offspring susceptibility to medulloblastoma induced by neonatal irradiation. This effect gained further biological significance thanks to a number of supporting data on the immunohistochemical characterization of the target tissue and preneoplastic lesions (PNLs). These results altogether pointed to increased proliferation of cerebellar granule cell precursors and PNLs cells, which favoured the development of frank tumours. The LOH analysis of tumor DNA showed Ptch1 biallelic loss in all tumor samples, suggesting that mechanisms other than interstitial deletions, typical of radiation-induced medulloblastoma, did not account for the observed increased cancer risk. This data was supported by comet analysis showing no differences in DNA damage induction and repair in cerebellar cells as a function of paternal irradiation. Finally, we provide biological plausibility to our results offering evidence of a possible epigenetic mechanism of inheritance based on radiation-induced changes of the microRNA profile of paternal sperm.

## INTRODUCTION

Several lines of evidence recently supported the notion of transgenerational inheritance not mediated by DNA sequence changes. Both genetic and environmental factors were shown to modulate this form of epigenetic inheritance, which involved complex metabolic, developmental and behavioral phenotypes. DNA methylation, histone-mediated chromatin conformation, and RNA were suggested as the possible epigenetic molecular messengers carried by the gametes. In particular, a central role of sperm-borne RNA was recently demonstrated by a number of independent studies [1–6].

The hypothesis of transgenerational carcinogenesis has long been controversial [7] because of the lack of a conceptual frame alternative to Mendelian inheritance. The possibility that parental exposure to ionizing radiation could increase the spontaneous or induced rate of tumors in the progeny had been initially explored in the ‘80s by several experimental mouse studies. Some studies had shown an effect of paternal exposure to radiation of different qualities on the incidence of spontaneous tumors and on the susceptibility of the progeny to chemical or physical carcinogens [8–11]. However, these results had not always been confirmed by independent studies [12, 13] suggesting that variables other than paternal irradiation could have affected the experimental outcome [14]. An association between paternal radiation exposure and increased incidence of leukemia and lymphoma had been reported among young people near Sellafield nuclear plant [15], but the assessment of cancer incidence in the progeny of Hiroshima and Nagasaki survivors did not support a similar association [16]. Further epidemiological studies on occupational [17] and diagnostic [18] radiation exposure did not obtain conclusive evidence on an effect of paternal exposure.

Recently, the new discoveries on untargeted radiation response [19] provided a possible mechanistic explanation and biological plausibility to transgenerational radiation effects prompting a new series of mouse studies on transgenerational genomic instability. These studies showed increased rates of DNA damage and mutations at repetitive and non-repetitive DNA sequences after paternal irradiation [20–22]. Germ cells differentiate in male mammals through spermatogenesis, a complex developmental process whose stages respond differentially to ionizing radiation [23]. Regarding male-mediated transgenerational carcinogenesis different stages were shown to be possible targets [10]. However, two of the most sound papers about transgenerationally induced genomic instability pointed to spermatogonia as a sensitive stage [21, 22]. Changes of DNA methylation levels and methyltransferase expression in the progeny of irradiated male mice have been shown in association to increased levels of DNA damage [24] supporting an epigenetic mechanism for transgenerational effects [25]. In humans, radiation-induced transgenerational genomic instability has been reported [26], but other studies did not find evidence of this phenomenon [27].

Taking advantage of the new mechanistic understanding about epigenetic inheritance, we decided to further test the hypothesis that irradiation of spermatogonia could increase cancer susceptibility in the F1 progeny using *Ptch1^+/−^* mice as a tumor-susceptible experimental model. These mice, heterozygous for a null mutation of the *Patched1* gene, are prone to develop spontaneous medulloblastoma, with a rate that is strongly increased by neonatal irradiation [28]. The physiology of cerebellum differentiation in the neonatal mouse is well known, from the proliferation of external granule layer (EGL) precursor cells to their internal migration and differentiation into postmitotic neurons. In EGL cells, the transmembrane protein Patched1 is an important regulator of this process through its binding Sonic hedgehog (Shh), a soluble extracellular factor secreted by Purkinje cells underneath the EGL [29].

Further to the demonstration of enhanced susceptibility to radiation-induced medulloblastoma in the progeny of irradiated male mice, we characterized the microRNA (miRNA) profile of paternal spermatozoa to investigate whether irradiation changed the content of this hypothetical epigenetic transgenerational messenger.

## RESULTS

Table 1 reports a synopsis of the analyses carried out in the parental and F1 generation.

**Table 1:** Synopsis of the analyses carried out in the parental and F1 generation

*Parental generation*	
6 weeks post irradiation	Sperm number
	Sperm miRNA profile
*F1 generation conceived 6 weeks after paternal irradiation*	
0–100 weeks after birth	Medulloblastoma induction
2 days after birth	DNA damage and repair in cerebellar cells
2 weeks after birth	GCP proliferation
4 weeks after birth	Evolution of preneoplastic lesions
8 weeks after birth	Evolution of preneoplastic lesions
3 months after birth	DNA damage in spleen and bone marrow cells

After mating, the males were sacrificed and their epididymal sperm number was assessed as a measure of the level of cytotoxicity induced by 1 Gy x-rays upon their progenitor spermatogonia. A slight but significant (*P* < 0.05) decrease of the sperm number in irradiated fathers (25.3 ± 1.8 vs. 36.1 ± 3.8 × 106) was detected, which, however, had no consequences on the number of pregnancies or on the average number of pups per litter (data not shown).

### Medulloblastoma induction

**Figure 1:**
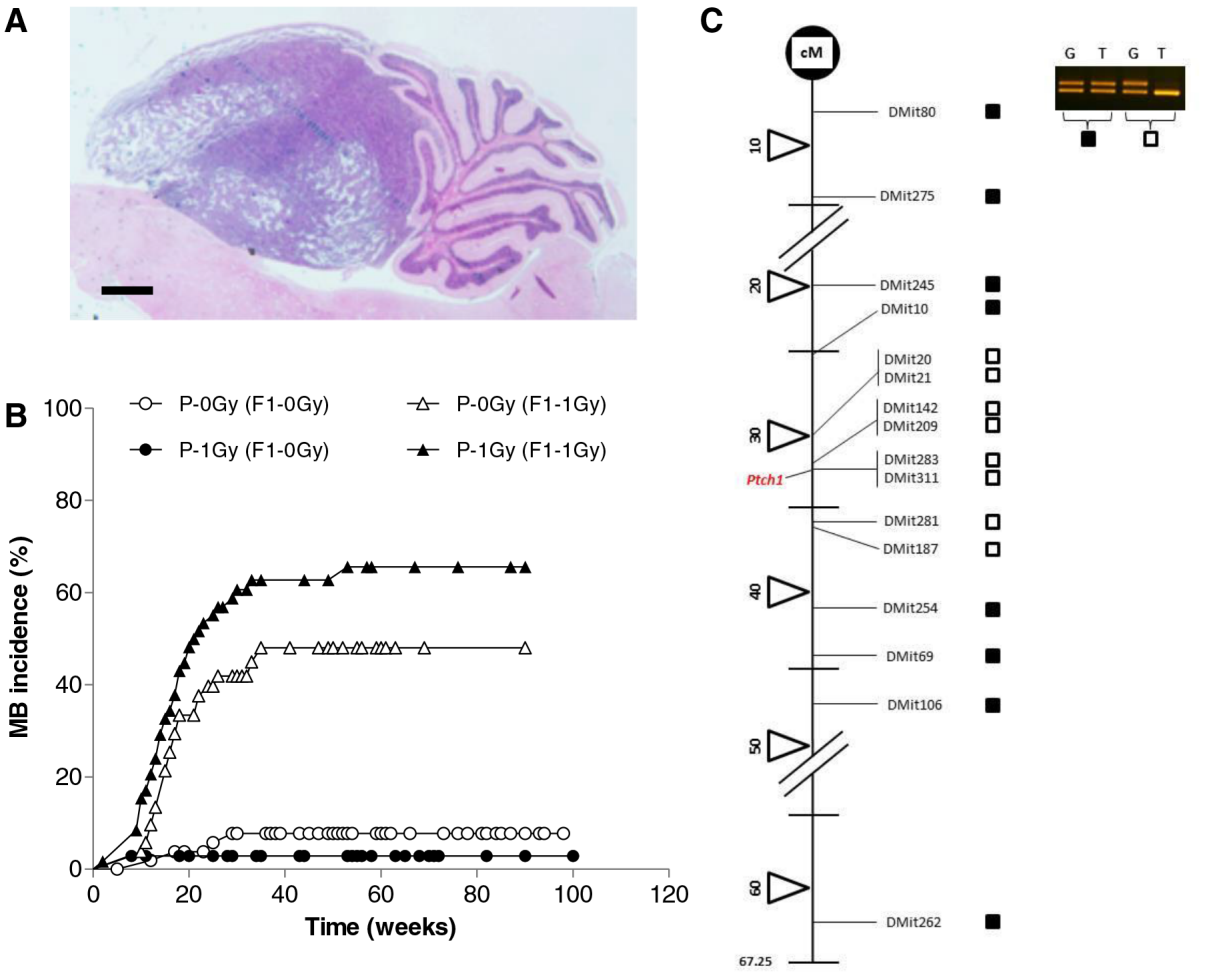
Medulloblastoma induction **A.** Representative histological image of medulloblastoma. Bar = 1000 μm. **B.** Kaplan-Meier kinetic analysis of medulloblastoma incidence obtained in unirradiated or P2-irradiated progeny of unirradiated [P-0Gy (F1–0Gy) and P-0Gy (F1–1Gy)] and irradiated fathers [P-1Gy (F1–0Gy) and P-1Gy (F1–1Gy)]. **C.** Representative analysis of chromosome-13 LOH in radiation-induced medulloblastomas. Solid squares indicate no LOH; open squares denote loss of signal from one allele (LOH); cM = centiMorgan.

To verify whether a paternal exposure to radiation is able to modify the spontaneous rate of this tumor in this mouse model, the progeny generated by wild-type male mice (unirradiated or irradiated with 1 Gy of x-rays) mated with *Ptch1^+/−^* female mice, was monitored for lifelong tumor development. Brain tumors were histopathologically examined and classified as medulloblastoma according to both location and morphological criteria (Fig. 1A). Results showed no significant difference (*P* = 0.1415) in spontaneous tumor development between groups, with a cumulative tumor incidence of 8.42% (8/95) and 2.94% (2/68) in P-0Gy (F1–0Gy) and P-1Gy (F1–0Gy) mice, respectively (Fig. 1B). In the P-1Gy (F1–0Gy) group, the median time to tumor onset was 8.5 weeks compared with 14.5 weeks of the P-0Gy (F1–0Gy) mice. However, this difference was not statistically significant. Therefore, we monitored tumor development in postnatal day 2 (P2) irradiated progeny (dose = 1 Gy) of unirradiated or irradiated fathers. As shown in Figure 1B, in P-0Gy (F1–1Gy) group, 23/52 (44%) mice developed medulloblastomas with a median time to tumor onset of 17.6 weeks (Fig. 1B). Importantly, we observed a borderline statistically significant increase of medulloblastoma rate (37/59; 63%) in P-1Gy (F1–1Gy) group (*P* = 0.0573), with the same latency of their counterpart (17.1 weeks).

Then, we analyzed medulloblastomas for allelic imbalance at the *Ptch1* locus, being the biallelic *Ptch1* loss the major pathway to medulloblastoma development in this mouse model [30]. Medulloblastomas from P-0Gy (F1–1Gy) (*n* = 18) or P-1Gy (F1–1Gy) mice (*n* = 27) were analyzed using a minimum of 16 informative microsatellite markers spanning mouse chr 13. All tumors showed a chr-13 interstitial deletion between 30–36 cM (Fig. 1C), including the *Ptch1* gene that is located at 32.8 cM according to the Mouse Chromosome 13 Linkage Map (http://www.informatics.jax.org/).

### Short-term analyses in cerebellum of F1 mice

#### DNA damage induction and repair

To verify whether paternal irradiation can induce in the F1 progeny a phenotype of genomic instability [21], possibly predisposing to tumorigenesis, satellite groups of F1 mice, progeny of irradiated or unirradiated fathers, were sacrificed at P2 to evaluate DNA damage in cerebellar cells by comet assay. Endogenous DNA damage was assessed in unirradiated newborns. Radiosensitivity and repair capacity were evaluated immediately and 1 hour after irradiation, respectively. The sensitivity of the method was tested by a preliminary experiment determining the dose-effect relationship.

**Figure 2:**
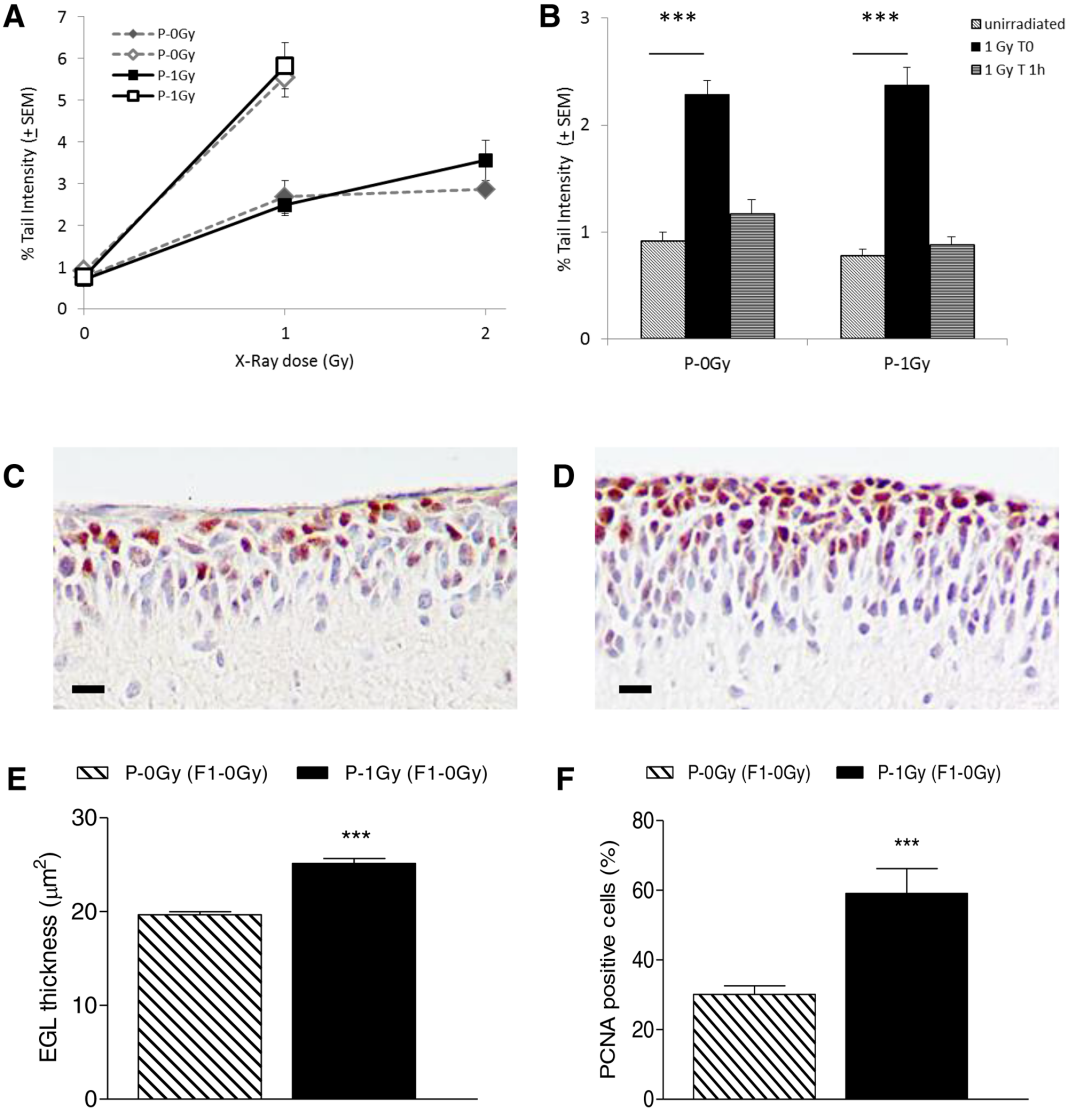
Comet assay and immunohistochemistry results in cerebellum cells from P2 mice **A.** Dose-response curves for comet tail intensity in cerebellum cells from irradiated offsprings of irradiated (P-1Gy) or unirradiated (P-0Gy) fathers. On the same graphic, open symbols represent Tail Intensity values in cells irradiated with 1Gy x-ray on slides. Tail Intensity values were statistically increased in all irradiated groups over matched unirradiated groups (*P* < 0.0001). **B.** DNA repair in the progeny of irradiated (P-1Gy) or unirradiated (P-0Gy) fathers. Induced damage (1 Gy T0, *P* < 0.0001) and residual damage 1 hour after irradiation (1 Gy T 1 h) are shown. **C–D.** Representative image of EGL immunostained with anti-PCNA antibody from 2-weeks old unirradiated progeny of non-irradiated (C) and irradiated fathers (D) **E.** Rate of GCPs proliferation index, expressed as percentage of PCNA positive cells over the total number of cells counted in each mouse cerebellum, and **F.** relative EGL thickness (*n* = 6). *P* < 0.0001. Bars = 10 μm.

The results showed an equal background level of DNA damage and a similar sensitivity toward irradiation in the progeny of irradiated and unirradiated fathers (Fig. 2A). Also the protocol modified to enhance the assay sensitivity by irradiation of agar-embedded cells did not evidence differences in the level of radiation-induced DNA damage between the two progeny groups. Similarly, no differences were shown in the residual level of DNA damage 1 hour after irradiation (Figure 2B). The same results were obtained when data were analyzed irrespectively of *Ptch1* genotype as reported in the figure, or when data were disaggregated by genotype (data not shown).

**Figure 3:**
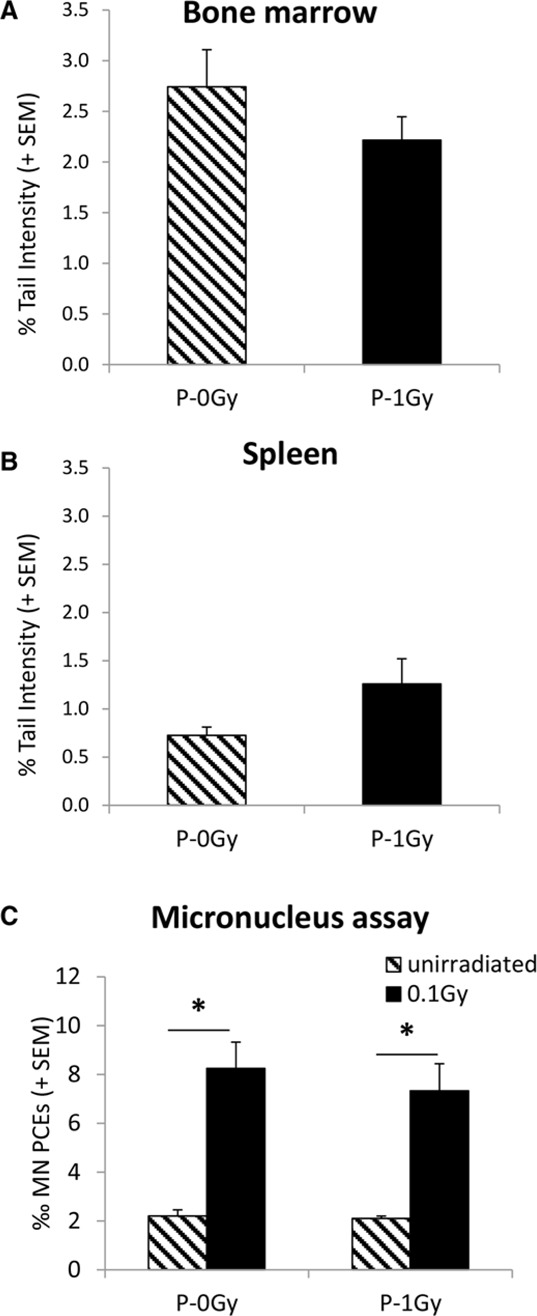
Genomic instability in adult progeny of irradiated (P-1Gy) or unirradiated (P-0Gy) fathers Background levels of comet tail intensity in bone marrow **A.** and spleen **B.** cells. The comparison of Tail Intensities did not show any effect of parental irradiation on the endogenous level of DNA damage in these organs. Background and radiation-induced frequencies of bone marrow micronucleated PCEs **C.** confirmed no increase of spontaneous genomic instability due to paternal irradiation and demonstrated that sensitivity to radiation-induced chromosome damage was similarly unaffected. * = *P* < 0.005

Consistently with these results, no evidence of increased spontaneous or radiation-induced genomic instability was shown in bone marrow and spleen of adult progeny of irradiated fathers (Fig. 3).

#### Proliferation and differentiation of cerebellar granule cell precursors

To test the hypothesis that paternal irradiation could alter the transition between proliferation and differentiation in GCPs, we measured EGL thickness and GCPs proliferative index in the 2-weeks old unirradiated progeny, of irradiated- and non-irradiated fathers (Fig. 2C–E). A significant increase in EGL thickness and a concomitantly significantly higher percentage of PCNA positive cells were observed in P-1Gy (F1–0Gy) progeny compared with P-0Gy (F1–0Gy) counterpart (*P* < 0.0001), suggesting that paternal irradiation had affected the proliferation signaling pathway of target tissue.

### The fate of medulloblastoma preneoplastic lesions

**Figure 4:**
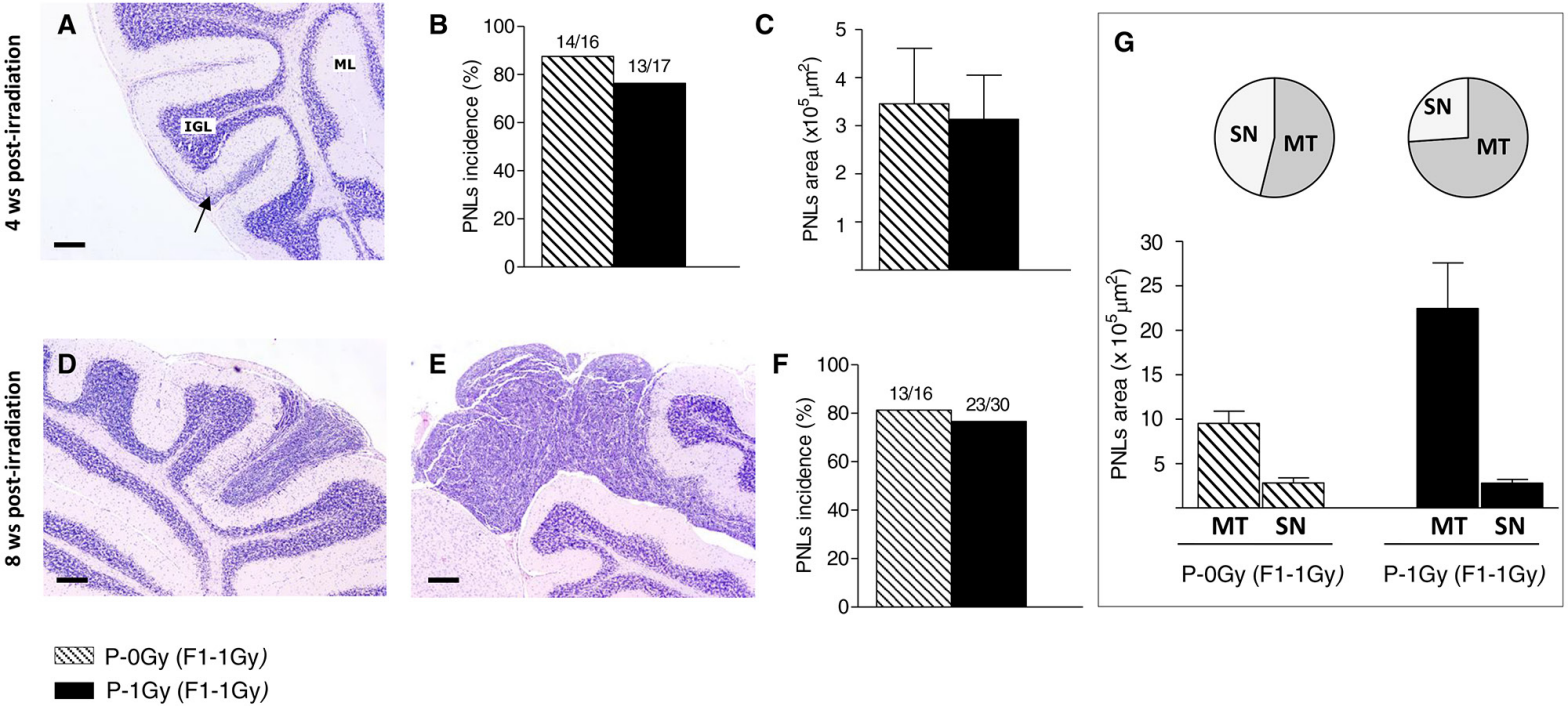
Medulloblastoma preneoplastic lesions in the irradiated progeny of unirradiated and irradiated fathers **A–C.** Histologic appearance of PNLs (arrow) at 4 weeks of age, their incidence (B) and dimensional analysis (C) **D–G.** At 8 weeks of age, in the cerebella of irradiated-F1 progeny, PNLs appear as small nodules (D) or microtumors (E), with no difference in total incidence between groups (F) Graphic representation of PNLs distribution by size (pie charts) and average areas (histogram) in both experimental group; MT: microtumors, SN: small nodules (G) Bars = 200 μm.

To evaluate whether paternal irradiation could influence PNLs progression, we measured the incidence of PNLs and characterized their morphology at 4 and 8 weeks of age in the irradiated progeny of irradiated and unirradiated fathers. At 4 weeks of age, PNLs appeared as hyperproliferation areas (Fig. 4A). As shown in Figure 4B, in P-0Gy (F1–1Gy) group, 13/17 (77%) mice developed PNLs; a very similar incidence (87%) was observed in the P-1Gy (F1–1Gy) progeny (14/16). Moreover, the dimensional analysis carried out on positive mice of both groups showed no difference in the PNLs area (Fig. 4C).

**Figure 5:**
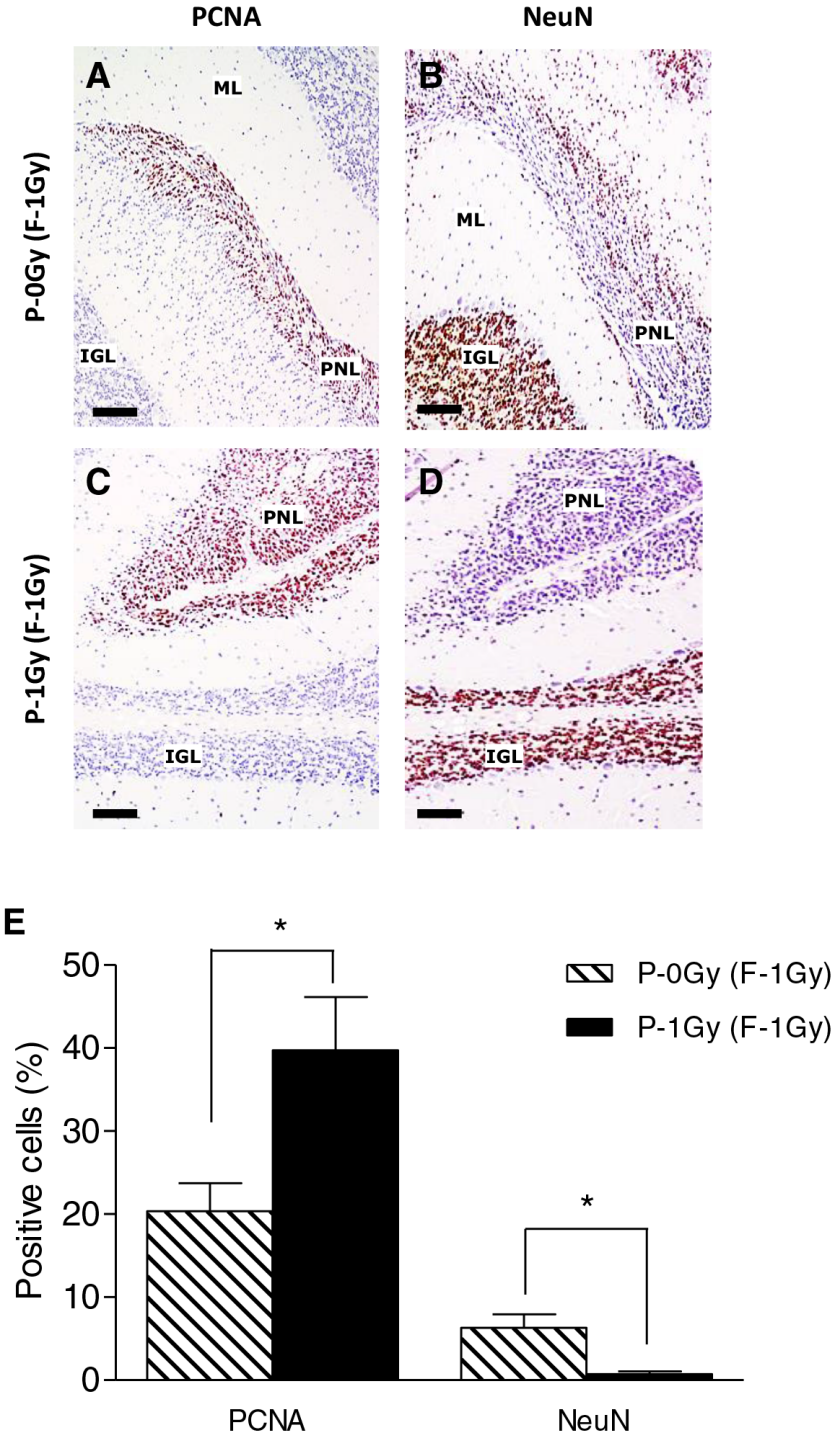
Paternal irradiation drives PNLs towards a more proliferative condition **A–D.** Representative images of PNLs (area < 5 × 10^5^ μm^2^) immunostained with anti-PCNA (A and C) and anti-NeuN (B and D). **E.** Immunohistochemical analysis showed significantly higher PCNA positive cells (*n* = 6; *P* < 0.05) along with lower NeuN expression (*P* < 0.05) in PNLs from irradiated fathers compared to unirradiated counterpart (*n* = 6). ML = Molecular layer; IGL = internal granule layer; PNL = Preneoplastic Lesion. Bars = 100 μm.

At 8 weeks of age, PNLs were classified according to histological and dimensional analysis as small nodules (area < 5 × 10^5^ μm^2^, Fig. 4D) or asymptomatic microtumors (area > 5 × 10^5^ μm^2^, Fig. 4E). Although the incidence of positive mice for cerebellum abnormalities was similar (Fig. 4F), a different size distribution of PNLs was observed between groups. As shown in Fig. 4G, in the P-1Gy (F1–1Gy) progeny, the relative frequency (17/23; 74%) and the average area of microtumors were higher compared with the P-0Gy (F1–1Gy) group (7/13; 53%), suggesting that, in the progeny of irradiated fathers, the progression of cerebellum abnormalities was accelerated. In addition, we characterized small nodules for markers of proliferation/differentiation to investigate their propensity to develop into medulloblastoma or to regress to normal tissue and to evaluate the influence of paternal irradiation on their fate. Serial sections of each lesion were immunostained with an anti-PCNA antibody (Fig. 5A and 5C), and with an antibody against NeuN, a marker of neuronal differentiation (Fig. 5B and 5D). As reported in Fig. 5E, small nodules of P-0Gy (F1–1Gy) showed 20% of PCNA- and 6% of NeuN-positive cells. P-1Gy (F1–1Gy) nodules showed a significant increase of proliferating cells and a virtual absence of differentiated cells, suggesting a more likely commitment to tumor progression.

### Paternal sperm miRNA characterization

**Figure 6:**
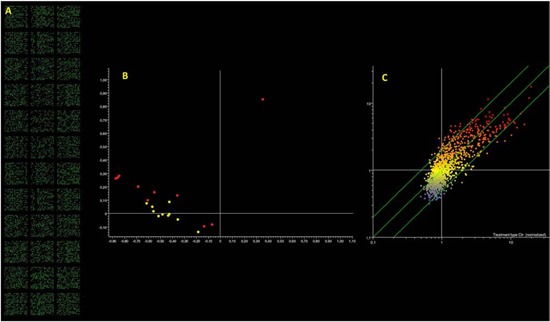
Microarray analysis of microRNA expression in paternal spermatozoa Panel **A.** fluorescent signal recorded indicating the high call-response rate despite the minimal microRNA amount used. Panel **B.** PCA results; each dot indicates the overall microRNA expression profile in a single mouse either unexposed (yellow dots) or exposed (red dots). Panel **C.** Scatter plot analysis; each dot indicates the expression intensity of a single microRNA in control (horizontal axis) and in radiation exposed mice (vertical axis). Dots located outside the 2-fold variation interval (diagonal green lines) are altered by treatment. Colors indicate the level of expression of each microRNA (blue, low; yellow intermediate; red, high).

After showing an enhanced susceptibility to medulloblastoma in the progeny of irradiated male mice, unexplained by Mendelian inheritance, we explored the involvement of sperm-borne miRNAs in this phenomenon. The total amount of RNA extracted for each sample was 289.69 ± 25.01 ng (mean ± SE). Despite this limited amount of total RNA, microarray analyses produced reliable results as indicated by the response score (i.e., the percentage of hybridized spots) >90% (Fig. 6A). This finding indicates that the contribution of miRNA to total RNA was very high, which is typical of spermatozoa, in these cells the miRNA/RNA ratio being higher than in somatic cells [31].

Principal component analysis of microarray data reveals that samples collected from animals, which underwent radiation exposure, present a different microRNA expression profile as compared to unexposed controls (Fig. 6B). This situation is confirmed by scatter plot analysis (Fig. 6C) revealing that 63 miRNAs were modified more than 2-fold as compared to the controls by treatment. Volcano plot analyses (taking into account both fold variation and statistical significance, *P* < 0.05) show that 44 miRNAs (3.9% of tested miRNAs) fall outside the 2-fold variation interval, 28 (2.5%) being upregulated and 16 (1.4%) downregulated. The list of these miRNAs is reported in Table 2.

**Table 2:** MicroRNA altered by radiation exposure in mice progeny spermatozoa as detected by microarray and volcano-plot analyses

microRNA	Fold variation (Exposed/Control)	Biological function
miR-29c*	0.47 ↓	role in spermatogenesis; induce apoptosis through increasing Bax/Bcl-2 ratio
miR-30c	2.63 ↑	regulate myogenic differentiation
miR-30e	2.34 ↑	induce apoptosis
miR34c*	2.00 ↑	role in spermatogenesis
miR-92a	0.48 ↑	prevent endothelial dysfunction in mice
miR-98	2.29 ↑	involved in rat embryo implantation; inhibit cell proliferation
miR-99b	2.08 ↑	promote apoptosis
miR-122*	2.24 ↑	role in spermatogenesis
miR-124	3.33 ↑	tumour suppressor; regulate glucocorticoid sensitivity
miR-135a*	2.04 ↑	role in spermatogenesis
miR-144	0.41 ↓	targets PTEN
miR-145	2.01 ↑	tumour suppressor
miR-147	2.43 ↑	regulate murine macrophage inflammatory responses
miR-181a	0.46 ↓	promote osteoclast survival
miR-183	3.45 ↑	promote cell proliferation
miR-196a	2.14 ↑	oncomir
miR-326	0.45 ↓	tumour suppressor; regulate dopamine receptor D2 expression
miR-338	0.48 ↓	regulate osteogenic differentiation in mice
miR-341	0.45 ↓	involved in early development of mice
miR-346	2.02 ↑	involved in immune response
miR-361	2.04 ↑	tumour suppressor
miR-363	2.42 ↑	regulate endothelial cell properties and their communication with hematopoietic precursor cells
miR-425	3.03 ↑	regulate production of atrial natriuretic peptide
miR-433	2.83 ↑	inhibit cell migration and proliferation
miR-449c*	2.01 ↑	role in spermatogenesis
miR-450a	2.63 ↑	Not available
miR-465a	2.46 ↑	involved in ovarian development, folliculogenesis and female fertility
miR-466a	0.45 ↓	role in osmoregulation through Nfat5
miR-466f	0.33 ↓	role in osmoregulation through Nfat5; pro-apoptotic role
miR-466 h	0.34 ↓	“
miR-466i	0.36 ↓	“
miR-466q	0.36 ↓	“
miR-471	0.49 ↓	Not available
miR-500	2.12 ↑	liver development
miR-503	2.33 ↑	regulate osteoclastogenesis by targeting RANK
miR-532	0.40 ↓	pro-apoptotic role
miR-666	0.44 ↓	mediate osmolar changes via aquaporin-1
miR-669d	0.48 ↓	epigenetic regulators of renal Nfat5 signaling
miR-669f	0.34 ↓	involved in helper T cell hyper-proliferation
miR-669g	2.18 ↑	Not available
miR-701	2.16 ↑	Not available
miR-713	2.12 ↑	Not available
miR-714	2.05 ↑	Not available
miR-762	2.24 ↑	involved in immune response

* miRNA involved in spermatogenesis

↑ upregulated

↓ downregulated

## DISCUSSION

The hypothesis that paternal exposure to ionizing radiation could transmit to the progeny a trait of increased cancer susceptibility was initially raised by experimental studies [10], but the issue remained unresolved largely because a mechanistic understanding of inheritance, alternative to Mendelian laws, was lacking [32]. An indirect support to the hypothesis of radiation-induced transgenerational carcinogenesis came from the evidence of transgenerational genomic instability induced in mice by spermatogonial irradiation, including data on dose and dose-rate dependent effectiveness [20–22]. Recently, the discoveries of untargeted radiation effects [19] and epigenetic forms of inheritance [33] provided a conceptual frame for the interpretation of transgenerational study results, paving the way to a reassessment of this phenomenon.

Here we report the results of a study in mice aimed at evaluating whether the progeny conceived by sperm descendant from irradiated spermatogonia showed increased incidence of spontaneous or radiation-induced medulloblastoma. We did not observe an elevated incidence of spontaneous tumors, but the progeny of irradiated fathers showed an increased frequency of medulloblastoma induced by neonatal irradiation. The cell-of-origin for medulloblastoma is the cerebellar granule cell precursor (GCP). In the first 3 weeks after birth, these cells actively proliferate in the external granule cell layer (EGL) of the cerebellum, then, postmitotic granule cells differentiate, and descend along Bergmann glia, to form the internal granule cell layer (IGL) [34]. We showed that paternal irradiation perturbed this process. In fact, the EGL thickness and the percentage of PCNA-positive GCPs were higher in the 14d-old progeny of irradiated than of unirradiated fathers. These results suggest that paternally inherited factors either delayed the differentiation or increased the proliferation rate of GCPs in the neonatal cerebellum. Although this effect was insufficient to trigger spontaneous tumor formation, the progeny of irradiated fathers showed an increased frequency of medulloblastoma induced by neonatal irradiation. Although borderline statistically significant, we interpreted the 1.4-fold increase of tumor incidence as very likely due to paternal irradiation, especially considering the expected inter-individual variability of mice in the radiation response and in the probability of transmission of any epigenetic effector to the following generation. To strengthen this result, we extended our study to the characterization of the early development phase of MB. In fact, one remarkable feature of radio-induced medulloblastoma in *Ptch1^+/−^* mice is the development through microscopically recognizable preneoplastic lesions (PNLs), ranging from small areas of hyperproliferation of granule neurons to overt nodules, and finally to microtumors. Only a subset of PNLs eventually progress to medulloblastoma [30]. Consistently with the alterations observed in the unirradiated target tissue at 14 days of age, 8 weeks after irradiation we observed a shift in the regression/progression balance in PNLs evolution enhancing their propensity to develop into medulloblastoma. This higher commitment to tumor progression strongly support the biological relevance of the 40% increase of cancer susceptibility obtained in the long-term carcinogenesis study.

The LOH analysis of tumor DNA showed *Ptch1* biallelic loss in all tumor samples. This data indicated that mechanisms other than interstitial deletions, typical of radiation-induced medulloblastoma, had not to be invoked to explain the observed increased cancer risk. For this reason, experiments were conducted to comparatively assess in the progeny of irradiated and unirradiated mice the level of DNA damage induction and repair in cerebellar cells. No evidence of a paternal radiation effect was obtained either for the basal and radiation induced DNA damage or for its repair, suggesting that the increased induction of *Ptch1* deletions could not be ascribed to higher levels of DNA damage or alterations of repair kinetics. Consistently with data on cerebellar cells, no increase of DNA damage was shown by comet and micronucleus tests in the irradiated father progeny bone marrow and spleen. Other authors [21] had shown evidence of genomic instability in these organs after comparable paternal irradiation conditions; this inconsistency could be reconciled considering the difference in mouse strains between the experiments.

Recently, several independent studies [35–37] supported the existence of epigenetic mechanisms of transgenerational inheritance affecting complex behavioural, developmental and metabolic traits. The molecular effectors and pathways of male-mediated epigenetic inheritance are still under investigation, but both noncoding RNAs [1–6] and DNA methylation [38] seem to play a role. Recently, the human sperm full RNA repertoire has been characterized, showing its complexity including rRNA, mRNA and both large and small non-coding RNAs [39, 40]. Mouse spermatozoa have been shown to deliver a unique set of miRNAs to the fertilized embryos, which are not present in the oocyte [41]. A functional role of some of these RNAs is emerging, as demonstrated by the arrest of preimplantation embryonic development in the absence of miRNA34c [41]. The strongest evidence for a functional role of sperm RNA in the transmission of non-Mendelian epigenetic inheritance comes from the experiments in which injection of specific RNAs into the fertilized egg leads to the reproducible induction of phenotypes ranging from the white tail *Kit*-mediated trait [1, 6], to cardiac hypertrophy [2], prenatal and postnatal overgrowth [3], and stress-induced behavioral and metabolic alterations [5]. An impact of environment on RNA-mediated epigenetic inheritance has been also demonstrated by metabolic alterations in the offspring of high fat-diet treated male mice associated to changes in paternal sperm miRNA content [4].

In this work we demonstrate for the first time that irradiation of premeiotic male germ cells leaves an epigenetic signature in the mature gametes: 44 out of 1135 analyzed miRNAs were differentially expressed in the sperm descendant from irradiated or unirradiated spermatogonia. From a functional standpoint, according to PubMed (http://www.ncbi.com) literature, these miRNAs are involved in multiple pathways, including regulation of cell death, neoplastic transformation and spermatogenesis (Table 2). Our experiment cannot establish a direct cause-effect relationship between variation of sperm miRNA profile and susceptibility to radiation-induced medulloblastoma in the progeny, neither can it point to one or more of the differentially expressed miRNA as mediator(s) of the transgenerational effect. This is a complex issue since specific epigenetic changes in sperm do not necessarily need to be maintained in the offspring target tissue for the epigenetic inheritance to be expressed [35, 38], because, during development, multiple layers of epigenetic control may transduce the original intergenerational message. In view of the emerging role of RNA in epigenetic inheritance described above, our results indicate a promising direction for future studies aiming at understanding the mechanisms of inherited susceptibility to radiation-induced medulloblastoma.

In this study we showed that paternal irradiation did not influence the spontaneous rate of medulloblastoma in the progeny, but synergistically with a neonatal radiation stress increased the tumor incidence. Causes of childhood cancer are not completely elucidated. Showing an influence of preconceptional paternal exposure to environmental stressors, in addition to the more understandable role of prenatal and neonatal exposures, might have an impact on disease prevention.

## MATERIALS AND METHODS

### Mice

A colony of *Ptch1^+/−^* mice, maintained on CD1 background, was obtained in our animal facility and genotyped as described [42]. Mice were housed under conventional conditions with food and water available ad libitum and 12-hour light-dark cycle. Care of animals was in accordance with the national legislation on animal experimentation, and experimental protocols were reviewed by the Institutional Animal Care and Use Committee.

### Paternal irradiation

CD1 male mice, 60–90 days old, were irradiated with a single dose of 1 Gy of x-rays (P-1Gy) using a Gilardoni CHF 320 G x ray generator (Gilardoni, Mandello del Lario, Italy) operated at 250 kVp, 15 mA, with Half-Value Layer = 1.6 mm Cu (additional filtration of 2.0 mm Al and 0.5 mm Cu). Six weeks after irradiation, each male was mated with two unirradiated *Ptch1^+/−^* females to generate a F1 progeny. According to the well-known time schedule of mouse spermatogenesis [43], sperm engaged in fertilization had been exposed to radiation at the stage of proliferating spermatogonia. In parallel, age-matched CD1 unirradiated male mice (P-0Gy) were mated with the same modality. To assess irradiation-induced cytotoxicity in spermatogonia, males were sacrificed at the end of mating and the number of epididymal spermatozoa was measured.

### Long-term carcinogenesis

F1 mice, born from 10 irradiated or 10 unirradiated males, were subdivided in two experimental groups. One group was irradiated with a single dose of 1 Gy (F1–1Gy) at post-natal day 2 (P2); the other group was left untreated (F1–0Gy). After genotyping, heterozygous F1 *Ptch1^+/−^* mice of both sexes were observed daily, and at the first sign of morbidity, were sacrificed and autopsied. Normally-appearing and tumor-bearing brains were fixed in 4% buffered formalin, embedded in paraffin wax according to standard techniques, sectioned and stained with hematoxylin/eosin for histology, or stored at −80°C for molecular analysis.

### Analysis of chromosome-13 LOH in medulloblastomas

DNA was extracted from frozen tissue using the Wizard SV Genomic DNA Purification System (Promega Corporation, Madison, WI). Because mice were genetically heterogeneous, we tested allelic polymorphisms for each microsatellite marker (D13Mit) using PCR primers for chromosome 13 (Research Genetics, Huntsville, Al, USA). The distance of D13Mit from the centromere is given in cM, with values taken from the Chromosome 13 Linkage Map of Mouse Genome Informatics (http://www.informatics.jax.org). A minimum of 16 informative D13Mit, spanning the length of chromosome 13, was used to examine medulloblastomas from all experimental groups. The experiment also involved analysis of genomic DNA from normal tissue as control.

### Immunohistochemical characterization of tumor target tissue and preneoplastic lesions

Additional groups of F1 mice were euthanized at different weeks of age to evaluate the External Granule Layer (EGL) thickness (2 weeks) or medulloblastoma preneoplastic lesions (PNLs) development (4 and 8 weeks). The entire cerebellum was examined recovering sections with intervals of 70 μm. The PNLs incidence was expressed as the percentage of mice with abnormal cerebellar regions with morphology varying from focal subpial aggregates of cerebellar granule cell precursors (GCPs) to overt nodules or microtumors. Morphometric analyses to measure EGL thickness or PNLs cross sectional areas were carried out using imaging software NIS-Elements BR 4.00.05 (Nikon Instruments Europe B.V., Italy).

Brain sections were cut at 4-μm thickness for immunohistochemical analysis of PCNA (monoclonal 1:100; Millipore, Billerica, MA, USA) and NeuN (monoclonal 1:100; Millipore) performed using the HistoMouse MAX Kit (Invitrogen Corporation, Camarillo, CA, USA) according to manufacturer's instructions. Blind immunohistochemical scoring was carried out and the percentage of positive (brown stained) cells was determined over the total number of cells counted in each sample.

### Alkaline comet assay on cerebellum cells

DNA damage was assessed on additional groups of at least 6 mice each. At P2, groups of mice were irradiated with a single dose of 1 or 2 Gy of x-rays; the spontaneous level of DNA damage was evaluated on groups of unirradiated mice. Mice were genotyped retrospectively. Immediately or one hour after irradiation, mice were euthanized and cell suspensions were obtained from cerebella. The assay was performed according to [44]. One-hundred cells from two different slides were analyzed by an image analysis system (Delta Sistemi, Rome, Italy). To evaluate the amount of DNA damage, Tail Intensity values were used. A modified version of the comet assay was also applied to increase its sensitivity by minimizing DNA repair of induced damage prior to cell lysis [45]. Briefly, cells from the control group embedded in agarose and cooled on ice were irradiated on slides with 1 Gy x-rays and immediately subjected to the comet assay procedure.

### Genomic instability in bone marrow and spleen cells of adult progeny

#### Comet assay on bone marrow and spleen cells

Additional groups of F1 3-month old male mice, obtained as described before, were used to assess endogenous DNA damage in bone marrow cells and spleen leukocytes by alkaline comet assay. Bone marrow cells were flushed out from femurs and leukocytes were obtained by mincing the spleen and centrifuging the cell suspension on histopaque 1077 (Sigma-Aldrich, St. Louis, MO) according to standard protocol. Slides for comet assay were prepared and analyzed as for cerebellar cells.

#### Micronucleus (MN) test in bone marrow polychromatic erythrocytes (PCEs)

Additional groups of F1 3-month old male mice, obtained as described before, were used to assess endogenous and irradiation induced MN frequencies in bone marrow PCEs of *Ptch1^+/−^* mice. Parallel groups of mice, born from irradiated or unirradiated fathers, were divided in two subgroups: one was irradiated with 0.1 Gy x-rays and the other was left unirradiated. A challenging dose of 0.1 Gy was chosen to test the hypothesis that paternal irradiation could influence the frequency of radiation-induced micronucleus frequency in the progeny, because it was sufficient to induce a significant micronucleus increase [46], but low enough to maximize the probability to detect a differential response. Mice were sacrificed 24 hours after irradiation and bone marrow cell suspensions were smeared on slides and scored according to standard protocols. Micronucleus frequency was evaluated in 2000 PCEs per mouse.

### Sperm miRNA characterization

Total RNA was purified from spermatozoa of 10 irradiated and 10 unirradiated mice by using a commercially available kit (miRNeasy, Qiagen), with minor modifications. In brief, spermatozoa were first washed twice in PBS, then resuspended in a somatic cell lysis buffer (0.1% SDS, 0.5% Triton X-100 in H_2_O), to produce a suspension of essentially pure spermatozoa [39]. Cell concentrations were determined with a hemacytometer. Lysis buffer was added to the samples at 600 μL/10^7^ cells, then the lysates were homogenized with a 26-gauge needle and heated for 30 minutes at 65°C. After incubation, the samples were homogenized a final time to ensure shearing of the DNA, loaded onto the commercial column, digested with RNase-free DNase I, and processed as described by the manufacturer. Total RNA was eluted from the column with 50-μL of RNase-free H_2_O. The amount and purity of the extracted RNA were evaluated by fiber optic spectrophotometer (Nanodrop ND-1000) calculating the 260/230 and 260/280 absorbance ratios.

miRNAs expression was analyzed using the 7th generation miRCURY LNATM microRNA Array (Exiqon), which contains 1,135 mouse miRNA probes. The total RNA from each sample was labeled by Cy3/Cy5 with Label IT^®^ miRNA Labeling Kits, Version 2 (Mirus Bio, WI). Samples, purified onto a chromatographic column and added with Hybridization Solution (EXIQON, Vedbaek, Denmark), were transferred to the microarray and covered with coverslips. The hybridization was performed in GlassArray Hybridization Cassettes (Invitrogen Ltd, Paisley, UK) in a waterbath at 37°C for 16 h and then a wash sequence was performed. Microarrays were dried by centrifugation and analyzed by laser scanner (ScanArray, PerkinElmer, Waltham, MA).

The microarray data were processed by GeneSpring software and their variability, as related to treatment, examined by box-plot analysis, scatter-plot analysis, hierarchical cluster analysis, and principal component analysis (PCA). Individual miRNAs modulated by the experimental treatment were identified by volcano-plot analysis.

### Statistics

Analyses were performed using GraphPad Prism 5.0 for Windows (GraphPad Software, San Diego, CA, USA). Kaplan-Meier survival curves were compared, and long-rank test *P* values calculated. PNLs incidence was analyzed using Fisher's exact test. Immunohistochemical scoring, morphometric analyses, comet assay and micronucleus results are reported as means ± SEM, and *t* test was used for determination of statistical difference between groups.

The microarray data have been processed by GeneSpring software (Agilent Santa Clara, CA, USA) subtracting from spot signals the local background, log transformation of signal intensity, data normalization both per spot and per chip. MicroRNA overall variability, as related to treatments, was examined by scatter-plot analysis taking into account a 2-fold variation between exposed and non-exposed animals and by principal component analysis of variance (PCA). Individual miRNAs modulated by the experimental treatments have been identified by volcano-plot analysis, taking into account 2-fold variation and *P* < 0.05 as evaluated by ANOVA on replicated data.
